# Piperazine-1,4-diium bis­(3,5-dicarboxy­benzoate)

**DOI:** 10.1107/S1600536810013140

**Published:** 2010-04-17

**Authors:** Gui-Ying Dong, Li-Hua Fan, Li-Xia Yang, Islam Ullah Khan

**Affiliations:** a College of Chemical Engineering and Biotechnology, Hebei Polytechnic University, Tangshan 063009, People’s Republic of China; b Materials Chemistry Laboratory, Department of Chemistry, Government College University, Lahore 54000, Pakistan

## Abstract

The asymmetric unit of the title salt, C_4_H_12_N_2_^2+^·2C_9_H_5_O_6_^−^, comprises one half of the piperazine-1,4-diium dication lying on an inversion centre and one 3,5-dicarboxy­benzoate anion. In the crystal, the ions are linked into a two-dimensional framework parallel to (101) by N—H⋯O and O—H⋯O hydrogen bonds.

## Related literature

For related structures, see: Divya *et al.* (2003 ▶); Sharma & Zaworotko *et al.* (1996 ▶). 
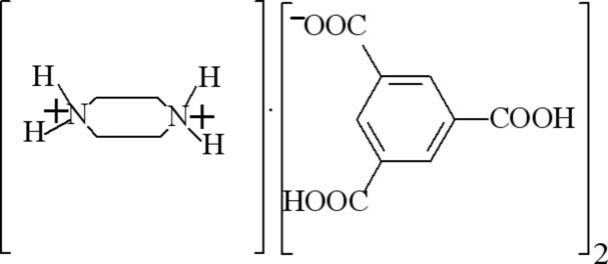


## Experimental

### 

#### Crystal data

C_4_H_12_N_2_^2+^·2C_9_H_5_O_6_^−^
*M* *_r_* = 506.42
Triclinic, 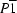

*a* = 7.3029 (15) Å
*b* = 8.6758 (17) Å
*c* = 9.0422 (18) Å
α = 87.04 (3)°
β = 69.94 (3)°
γ = 83.76 (3)°
*V* = 534.9 (2) Å^3^
*Z* = 1
Mo *K*α radiation
μ = 0.13 mm^−1^
*T* = 295 K
0.22 × 0.21 × 0.20 mm

#### Data collection

Bruker SMART CCD area-detector diffractometer
Absorption correction: multi-scan (*SADABS*; Sheldrick, 1996 ▶) *T*_min_ = 0.968, *T*_max_ = 0.971
5586 measured reflections
2443 independent reflections
1563 reflections with *I* > 2σ(*I*)
*R*_int_ = 0.048

#### Refinement

*R*[*F* ^2^ > 2σ(*F* ^2^)] = 0.069
*wR*(*F* ^2^) = 0.219
*S* = 1.00
2443 reflections
165 parameters
H-atom parameters constrained
Δρ_max_ = 0.35 e Å^−3^
Δρ_min_ = −0.22 e Å^−3^

### 

Data collection: *SMART* (Bruker, 1998 ▶); cell refinement: *SAINT* (Bruker, 1999 ▶); data reduction: *SAINT*; program(s) used to solve structure: *SHELXS97* (Sheldrick, 2008 ▶); program(s) used to refine structure: *SHELXL97* (Sheldrick, 2008 ▶); molecular graphics: *SHELXTL* (Sheldrick, 2008 ▶); software used to prepare material for publication: *SHELXTL*.

## Supplementary Material

Crystal structure: contains datablocks I, global. DOI: 10.1107/S1600536810013140/ci5078sup1.cif

Structure factors: contains datablocks I. DOI: 10.1107/S1600536810013140/ci5078Isup2.hkl

Additional supplementary materials:  crystallographic information; 3D view; checkCIF report

## Figures and Tables

**Table 1:** Hydrogen-bond geometry (Å, °)

*D*—H⋯*A*	*D*—H	H⋯*A*	*D*⋯*A*	*D*—H⋯*A*
N1—H1*A*⋯O4^i^	0.90	1.85	2.725 (4)	165
N1—H1*B*⋯O6^ii^	0.90	1.92	2.751 (4)	153
O2—H2⋯O4^iii^	0.82	1.87	2.612 (4)	149
O5—H5⋯O3^iv^	0.82	1.79	2.584 (4)	164

Symmetry codes: (i) 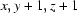; (ii) 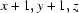; (iii) 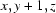; (iv) 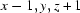.

**Fig. 1.**
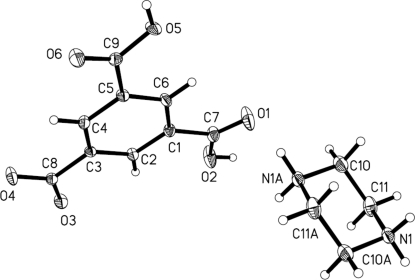
The structure of the title compound, showing the atomic numbering and 30% probability displacement ellipsoids. Atoms labelled with the suffix A are generated by the symmetry operation (1-x, 1-y, 1-z).

**Fig. 2.**
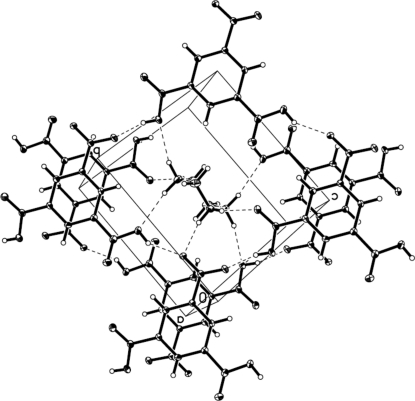
A partial packing diagram of the title compound. Hydrogen bonds are shown as dashed lines

## supplementary crystallographic information

### Comment

1,3,5-Benzenetricarboxylic acid is an important building block in crystal engineering due to its predictable honeycomb formation in the crystal lattice. It has six potential donor sites in the three carboxylic acid group, and it can form mono-, di- and trianionic ligand species through deprotonation. The adduct of 4,4'-bipyridyl with trimesic acid (1,3,5-benzenetricarboxylic acid) is of 2:3 stoichiometry and it forms a two-dimensional network (Sharma & Zaworotko, 1996). We report here the crystal structure of the title compound, (I).

The asymmetric unit of the compound (I) comprises one-half of a piperazine-1,4-diium cation which lies on an inversion centre and one 3,5-dicarboxy benzoate anion (Fig. 1). Bond distances and angles in (I) are normal (Divya *et al.*, 2003).

In the crystal structure, the cations and anions are interlinked by N—H···O and O—H···O hydrogen bonds (Table 1) producing a two-dimensional hydrogen-bonded framework structure parallel to the (101) [Fig. 2].

### Experimental

1,3,5-Benzenetricarboxylic acid (1.06 g, 5 mmol) and piperazine (0.43 g, 5 mmol) were dissolved in warm water (30 ml). Single crystals of the title compound were obtained by slow evaporation of this solution.

### Refinement

H atoms were positioned geometrically [O–H = 0.82 Å, N–H = 0.90 Å and C–H = 0.93 or 0.97 Å] and refined using a riding model, with Uiso(H) = 1.5U_eq_(O) and 1.2U_eq_(C,N).

### Crystal data

**Table:**

C_4_H_12_N_2_^2+^·2C_9_H_5_O_6_^−^	*Z* = 1
*M**_r_* = 506.42	*F*(000) = 264
Triclinic, *P*1	*D*_x_ = 1.572 Mg m^−^^3^
Hall symbol: -P 1	Mo *K*α radiation, λ = 0.71073 Å
*a* = 7.3029 (15) Å	Cell parameters from 2886 reflections
*b* = 8.6758 (17) Å	θ = 4.1–23.7°
*c* = 9.0422 (18) Å	µ = 0.13 mm^−^^1^
α = 87.04 (3)°	*T* = 295 K
β = 69.94 (3)°	Prism, colourless
γ = 83.76 (3)°	0.22 × 0.21 × 0.20 mm
*V* = 534.9 (2) Å^3^	

### Data collection

**Table:**

Bruker SMART CCD area-detector diffractometer	2443 independent reflections
Radiation source: fine-focus sealed tube	1563 reflections with *I* > 2σ(*I*)
graphite	*R*_int_ = 0.048
φ and ω scans	θ_max_ = 27.5°, θ_min_ = 3.1°
Absorption correction: multi-scan (*SADABS*; Sheldrick, 1996)	*h* = −9→9
*T*_min_ = 0.968, *T*_max_ = 0.971	*k* = −11→11
5586 measured reflections	*l* = −11→11

### Refinement

**Table:**

Refinement on *F*^2^	Primary atom site location: structure-invariant direct methods
Least-squares matrix: full	Secondary atom site location: difference Fourier map
*R*[*F*^2^ > 2σ(*F*^2^)] = 0.069	Hydrogen site location: inferred from neighbouring sites
*wR*(*F*^2^) = 0.219	H-atom parameters constrained
*S* = 1.00	*w* = 1/[σ^2^(*F*_o_^2^) + (0.1021*P*)^2^ + 0.7768*P*] where *P* = (*F*_o_^2^ + 2*F*_c_^2^)/3
2443 reflections	(Δ/σ)_max_ = 0.001
165 parameters	Δρ_max_ = 0.35 e Å^−^^3^
0 restraints	Δρ_min_ = −0.22 e Å^−^^3^

### Special details

**Table:**

Geometry. All esds (except the esd in the dihedral angle between two l.s. planes) are estimated using the full covariance matrix. The cell esds are taken into account individually in the estimation of esds in distances, angles and torsion angles; correlations between esds in cell parameters are only used when they are defined by crystal symmetry. An approximate (isotropic) treatment of cell esds is used for estimating esds involving l.s. planes.
Refinement. Refinement of F^2^ against ALL reflections. The weighted R-factor wR and goodness of fit S are based on F^2^, conventional R-factors R are based on F, with F set to zero for negative F^2^. The threshold expression of F^2^ > 2sigma(F^2^) is used only for calculating R-factors(gt) etc. and is not relevant to the choice of reflections for refinement. R-factors based on F^2^ are statistically about twice as large as those based on F, and R- factors based on ALL data will be even larger.

### Fractional atomic coordinates and isotropic or equivalent isotropic displacement parameters (Å^2^)

**Table:**

	*x*	*y*	*z*	*U*_iso_*/*U*_eq_	
O3	0.5054 (4)	−0.0789 (3)	−0.2891 (3)	0.0327 (7)	
O4	0.3840 (4)	−0.2898 (3)	−0.1607 (3)	0.0351 (7)	
O5	−0.2059 (4)	0.0124 (3)	0.4770 (3)	0.0299 (6)	
H5	−0.2853	−0.0331	0.5479	0.045*	
O6	−0.0820 (4)	−0.2283 (3)	0.3989 (3)	0.0401 (8)	
C6	0.0627 (5)	0.1419 (4)	0.2228 (4)	0.0244 (8)	
H6	−0.0104	0.2046	0.3073	0.029*	
O2	0.3069 (5)	0.4294 (3)	−0.0579 (3)	0.0507 (9)	
H2	0.3110	0.5232	−0.0547	0.076*	
C2	0.2932 (5)	0.1130 (4)	−0.0401 (4)	0.0213 (7)	
H2A	0.3740	0.1567	−0.1322	0.026*	
C3	0.2797 (5)	−0.0471 (4)	−0.0306 (4)	0.0200 (7)	
C5	0.0477 (5)	−0.0166 (4)	0.2327 (4)	0.0220 (7)	
C1	0.1875 (5)	0.2071 (4)	0.0863 (4)	0.0238 (8)	
C4	0.1576 (5)	−0.1116 (4)	0.1067 (4)	0.0231 (8)	
H4	0.1490	−0.2181	0.1146	0.028*	
C8	0.3998 (5)	−0.1456 (4)	−0.1710 (4)	0.0221 (7)	
O1	0.1355 (6)	0.4589 (3)	0.1961 (4)	0.0588 (10)	
C9	−0.0858 (5)	−0.0890 (4)	0.3773 (4)	0.0243 (8)	
C7	0.2045 (6)	0.3776 (4)	0.0825 (4)	0.0324 (9)	
N1	0.6111 (5)	0.5988 (3)	0.5510 (3)	0.0294 (7)	
H1A	0.5332	0.6181	0.6509	0.035*	
H1B	0.7184	0.6495	0.5311	0.035*	
C11	0.5066 (7)	0.6562 (4)	0.4430 (5)	0.0375 (10)	
H11A	0.5946	0.6439	0.3352	0.045*	
H11B	0.4656	0.7659	0.4602	0.045*	
C10	0.3299 (6)	0.5704 (4)	0.4671 (5)	0.0385 (10)	
H10A	0.2357	0.5906	0.5716	0.046*	
H10B	0.2682	0.6068	0.3907	0.046*	

### Atomic displacement parameters (Å^2^)

**Table:**

	*U*^11^	*U*^22^	*U*^33^	*U*^12^	*U*^13^	*U*^23^
O3	0.0350 (14)	0.0304 (14)	0.0214 (13)	−0.0079 (11)	0.0066 (11)	−0.0010 (10)
O4	0.0490 (17)	0.0175 (13)	0.0256 (14)	−0.0037 (12)	0.0047 (12)	−0.0024 (10)
O5	0.0265 (14)	0.0288 (14)	0.0223 (13)	−0.0038 (11)	0.0074 (10)	0.0010 (10)
O6	0.0459 (17)	0.0261 (14)	0.0318 (15)	−0.0108 (12)	0.0099 (13)	0.0006 (11)
C6	0.0257 (18)	0.0196 (16)	0.0219 (17)	−0.0002 (14)	−0.0002 (14)	−0.0049 (13)
O2	0.085 (2)	0.0214 (14)	0.0282 (15)	−0.0164 (15)	0.0054 (15)	0.0018 (11)
C2	0.0253 (18)	0.0196 (16)	0.0157 (15)	−0.0037 (14)	−0.0025 (13)	0.0021 (13)
C3	0.0183 (16)	0.0200 (16)	0.0189 (16)	−0.0021 (13)	−0.0027 (13)	−0.0005 (12)
C5	0.0229 (17)	0.0230 (17)	0.0162 (16)	−0.0035 (14)	−0.0015 (13)	0.0003 (13)
C1	0.0280 (18)	0.0199 (17)	0.0224 (17)	−0.0043 (14)	−0.0064 (14)	0.0000 (13)
C4	0.0268 (18)	0.0203 (16)	0.0207 (17)	−0.0048 (14)	−0.0052 (14)	−0.0014 (13)
C8	0.0220 (17)	0.0220 (17)	0.0198 (16)	−0.0035 (14)	−0.0034 (14)	−0.0008 (13)
O1	0.091 (3)	0.0257 (15)	0.0370 (17)	−0.0137 (16)	0.0105 (17)	−0.0093 (13)
C9	0.0232 (17)	0.0249 (18)	0.0208 (17)	−0.0078 (15)	−0.0010 (14)	0.0005 (14)
C7	0.041 (2)	0.0224 (18)	0.0285 (19)	−0.0098 (17)	−0.0025 (17)	0.0000 (15)
N1	0.0306 (17)	0.0261 (16)	0.0255 (16)	−0.0069 (13)	0.0001 (13)	−0.0063 (13)
C11	0.058 (3)	0.0202 (18)	0.033 (2)	−0.0037 (18)	−0.0124 (19)	−0.0006 (15)
C10	0.038 (2)	0.033 (2)	0.044 (2)	0.0074 (18)	−0.0150 (19)	−0.0115 (18)

### Geometric parameters (Å, °)

**Table:**

O3—C8	1.240 (4)	C5—C4	1.396 (5)
O4—C8	1.265 (4)	C5—C9	1.493 (4)
O5—C9	1.314 (4)	C1—C7	1.496 (5)
O5—H5	0.82	C4—H4	0.93
O6—C9	1.213 (4)	O1—C7	1.204 (5)
C6—C5	1.387 (5)	N1—C11	1.471 (5)
C6—C1	1.394 (5)	N1—C10^i^	1.485 (5)
C6—H6	0.93	N1—H1A	0.90
O2—C7	1.318 (4)	N1—H1B	0.90
O2—H2	0.8200	C11—C10	1.505 (6)
C2—C1	1.387 (5)	C11—H11A	0.97
C2—C3	1.399 (4)	C11—H11B	0.97
C2—H2A	0.93	C10—N1^i^	1.485 (5)
C3—C4	1.388 (5)	C10—H10A	0.97
C3—C8	1.516 (5)	C10—H10B	0.97
			
C9—O5—H5	109.5	O6—C9—C5	122.5 (3)
C5—C6—C1	120.0 (3)	O5—C9—C5	113.5 (3)
C5—C6—H6	120.0	O1—C7—O2	123.3 (3)
C1—C6—H6	120.0	O1—C7—C1	123.8 (3)
C7—O2—H2	109.5	O2—C7—C1	112.9 (3)
C1—C2—C3	120.7 (3)	C11—N1—C10^i^	111.1 (3)
C1—C2—H2A	119.6	C11—N1—H1A	109.4
C3—C2—H2A	119.6	C10^i^—N1—H1A	109.4
C4—C3—C2	119.4 (3)	C11—N1—H1B	109.4
C4—C3—C8	121.7 (3)	C10^i^—N1—H1B	109.4
C2—C3—C8	118.9 (3)	H1A—N1—H1B	108.0
C6—C5—C4	120.3 (3)	N1—C11—C10	111.5 (3)
C6—C5—C9	121.0 (3)	N1—C11—H11A	109.3
C4—C5—C9	118.7 (3)	C10—C11—H11A	109.3
C2—C1—C6	119.6 (3)	N1—C11—H11B	109.3
C2—C1—C7	122.1 (3)	C10—C11—H11B	109.3
C6—C1—C7	118.3 (3)	H11A—C11—H11B	108.0
C3—C4—C5	119.9 (3)	N1^i^—C10—C11	110.1 (3)
C3—C4—H4	120.0	N1^i^—C10—H10A	109.6
C5—C4—H4	120.0	C11—C10—H10A	109.6
O3—C8—O4	124.4 (3)	N1^i^—C10—H10B	109.6
O3—C8—C3	117.7 (3)	C11—C10—H10B	109.6
O4—C8—C3	117.9 (3)	H10A—C10—H10B	108.2
O6—C9—O5	124.0 (3)		

Symmetry codes: (i) −*x*+1, −*y*+1, −*z*+1.

### Hydrogen-bond geometry (Å, °)

**Table:**

*D*—H···*A*	*D*—H	H···*A*	*D*···*A*	*D*—H···*A*
N1—H1A···O4^ii^	0.90	1.85	2.725 (4)	165
N1—H1B···O6^iii^	0.90	1.92	2.751 (4)	153
O2—H2···O4^iv^	0.82	1.87	2.612 (4)	149
O5—H5···O3^v^	0.82	1.79	2.584 (4)	164

Symmetry codes: (ii) *x*, *y*+1, *z*+1; (iii) *x*+1, *y*+1, *z*; (iv) *x*, *y*+1, *z*; (v) *x*−1, *y*, *z*+1.
